# Investigation on submicron particle separation and deflection using tilted-angle standing surface acoustic wave microfluidics

**DOI:** 10.1016/j.heliyon.2024.e25042

**Published:** 2024-01-29

**Authors:** Tao Peng, Xiaodong Lin, Luming Li, Lei Huang, Bingyan Jiang, Yanwei Jia

**Affiliations:** a Zhuhai UM Science & Technology Research Institute, Zhuhai, China; b State Key Laboratory of High-Performance Complex Manufacturing, College of Mechanical and Electrical Engineering, Central South University, Changsha, 410083, China; c State Key Laboratory of Analog and Mixed-Signal VLSI, Institute of Microelectronics, University of Macau, Macau, China; d Faculty of Science and Technology – Electrical and Computer Engineering, University of Macau, Macau, China; e MoE Frontiers Science Center for Precision Oncology, University of Macau, Macau, China

**Keywords:** Submicron particles, Acoustic radiation, Microfluidic separation, Acoustofluidics

## Abstract

With the development of in vitro diagnostics, extracting submicron scale particles from mixed body fluids samples is crucial. In recent years, microfluidic separation has attracted much attention due to its high efficiency, label-free, and inexpensive nature. Among the microfluidic-based separation, the separation based on ultrasonic standing waves has gradually become a powerful tool. A microfluid environment containing a tilted-angle ultrasonic standing surface acoustic wave (taSSAW) field has been widely adapted and designed to separate submicron particles for biochemical applications. This paper investigated submicron particle defection in microfluidics using taSSAWs analytically. Particles with 0.1–1 μm diameters were analyzed under acoustic pressure, flow rate, tilted angle, and SSAW frequency. According to different acoustic radiation forces acting on the particles, the motion of large-diameter particles was more likely to deflect to the direction of the nodal lines. Decreasing the input flow rate or increasing acoustic pressure and acoustic wave frequency can improve particle deflection. The tilted angle can be optimized by analyzing the simulation results. Based on the simulation analysis, we experimentally showed the separation of polystyrene microspheres (100 nm) from the mixed particles and exosomes (30–150 nm) from human plasma. This research results can provide a certain reference for the practical design of bioparticle separation utilizing acoustofluidic devices.

## Highlights

• Submicron particle deflection in taSSAW microfluidic systems was analyzed.
• Particle deflection can be adjusted by acoustic and flow fields.
• The tilted angle can be optimized with analytical calculation.
• The taSSAW microfluidic chip can achieve high efficiency compared to other methods.

## 1 Introduction

Separating and purifying biological samples at the submicron scale, such as plasma and saliva, to obtain particles with specific characteristics are highly significant in biochemical analysis and clinical medicine [[1], [2], [3], [4]]. They are crucial for developing the next generation of liquid biopsy technology. For example, exosomes (30–150 nm) are cell secretions with abundant detectable biomarkers (such as protein, miRNA, and lipid), which have attracted continuous attention in the research field of cell communication [5,6], drug delivery [7], and molecular engineering [8,9]. Removing large-sized particles in a mixed solution at the submicron scale is usually necessary to obtain exosomes.

Current conventional isolation techniques for submicron scales, such as differentiation ultracentrifugation [10], have disadvantages such as time-consuming, expensive instruments dependence, low recovery and purity [11]. In recent years, numerous microfluidics-based extraction methods have been developed that utilize the principle of immunoaffinity separation based on antibody labeling [12] or achieve separation based on the physical properties. Physical property-based microfluidic separations include the deterministic lateral displacement method [13], viscoelastic-flow sorting [14], acoustofluidic separation [[15], [16], [17]], dielectrophoretic methods [18] or a combination of the principle mentioned above [19].

Among these, the ultrasonic wave-based methods, which have the advantages of high integration, label-free, high efficiency, and biocompatibility, have attracted wide attention. Standing surface acoustic wave (SSAW) microfluidic utilizes interdigital transducers (IDTs) to generate a stable standing acoustic field in the fluidic channel and achieves separation based on differences in particle size [[20], [21], [22]] or other physical properties [[23], [24], [25]]. SSAW microfluidic can be divided into two types based on the angle of IDT and microchannel, parallel [23,24] and tilt type [26]. The lateral deflection distance in parallel type is confined to 1/4 acoustic wavelength, which limits the throughput of the separation. Compared with the parallel type, the tilted type generates multiple pressure nodes and anti-nodes in the fluidic channel. The particles will experience a more considerable lateral deflection distance, making achieving high throughput and precision easier [27].

The taSSAW microfluidic has been widely applied to biological particle separation, such as bacteria [28], tumor cells [26,27], extracellular microvesicles [29], and exosomes [17,30]. To fully grasp the potential of taSSAW microfluidic devices, further analysis of particle deflection and development in the reliability of the available devices is mandatory, which can be achieved by a numerical and analytical study [[31], [32], [33]]. Numerical methods have been demonstrated for the reliable design of bioparticle separation microfluidic devices [[34], [35], [36], [37], [38]]. Wang. et al. [39] analyzed the motion of particles in taSSAW microfluidic chip, which provides a basis for understanding particle behavior in SSAW field. Wu. et al. [40] provided an enhanced method for taSSAW microfluidic design, which can help explain the influence of acoustic waves on particle motion. Some scholars [[41], [42], [43]] investigated particle separation in taSSAW microfluidic chips through finite element simulations, and these studies are advantageous to the chip design. In our recent research [44], we presented a numerical model considering viscous drag force and the acoustic radiation force for studying particle deflection in the taSSAW microfluidic chip, which could be used to optimize the design and better understand the mechanism of particle separation.

The above study provided practical guidance for the engineering of acoustofluidic separation chips. However, currently employed numerical models are generally time-consuming and require many computing resources to study particle separation in taSSAW microfluidic chip and optimize the design, hindering the broad application of this technique in bioseparations, and comprehensive optimization study on submicron particle separation using tilted-angle SSAW microfluidics is relatively lacking. For analytical modelling of taSSAW microfluidic devices, Ding. et al. [26] established an analytical model for predicting particle motion, which can guide for tumour cell separation. Liu et al. [45] summarized and classified particle motion in taSSAW microchannel based on analytical calculations. Han et al. [46] focused on optimizing sorting parameters based on experimental observations and analytical modelling.

Herein, we analyzed submicron particle defection in microfluidics containing taSSAWs using an analytical model. The influences of acoustic pressure, flow rate, and chip parameters on particle motion have been thoroughly studied using the theoretical modelling system. Particle manipulation and separation were achieved by studying the parameters involved in the taSSAW microfluidic chip through analytical models. With the instruction of these analyses, we have experimentally separated exosomes from biological samples and 100 nm polystyrene microspheres from particle mixtures. The results can provide a reference for the engineering design of exosome microfluidic separation devices.

## 2 Theoretical and experimental setup

### 2.1 Working principle of taSSAW microfluidic chip for bioparticle separation

A taSSAW microfluidic chip usually consists of a 128° YX lithium niobate piezoelectric substrate, interdigital transducers (IDTs), and a microfluidic chip. SSAW-based microfluidic enables separation based on the difference of size [[47], [48], [49]], compressibility [50], and density [51], and when used for exosome sorting, the process is usually achieved based on size differences [30].

**Fig. 1:**
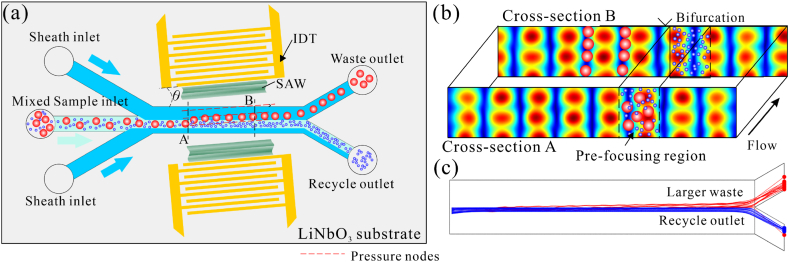
Principle of taSSAW microfluidic separation device. (a) Schematic illustration of the taSSAW microfluidic device. (b) The acoustic field distribution and particle in the cross-section of the microchannel. (c) The simulated results of particle trajectory.

Fig. 1a depicts the schematic view of the sized-based taSSAW microfluidic separation device. The operating principle is as follows: a pair of IDTs arranged in parallel generates an SSAW field, the acoustic waves leak into the microchannel arranged at an inclined angle *θ,* and periodic acoustic pressure distribution forms in the main channel. This study used the well-known sheath focusing process. The position and width of particle focusing can be controlled by the ratio of sheath flow to sample flow. When the prefocused particle mixture passes the acoustic field along the flow direction, since the acoustic radiation force (ARF) experienced by particles is proportional to the cube of the particle radius [50], the larger particles migrate toward the nodal lines under the influence of strong ARF and drag force, and finally flow to the waste outlet. In comparison, the smaller particles continue moving toward the recycle outlet due to the weak ARF. Based on the force difference and the unique arrangement, the trajectories of the larger and smaller particles are separated as they reach the bifurcation, and different sizes of particles with higher purity can be recycled. Fig. 1b shows the cross-section view of the acoustic field and particle distribution. Fig. 1c shows the trajectories of 500 and 100 nm particles in the taSSAW microfluidic channel.

### 2.2 Modeling of particle motion in taSSAW microfluidic device

In this study, we focus on the deflection of submicron particles in the taSSAW microfluidic device and the influence of acoustic and flow fields on particle motion. Because the motion of particles in the height direction did not affect the total separation, a detailed planer model was employed to analyze the particle motion and deflection. These assumptions have been considered to develop the governing equations in the mathematical model: (1) The fluid is homogeneous, and the interaction between particles and between particles and channel walls is not considered. (2) In the taSSAW microfluidic chip, particles experience ARF, drag force, gravitational and buoyant force. The gravitational and buoyant forces acting on particles do not affect the lateral deflection. Therefore, the gravitational and buoyant forces are ignored. (3) Acoustic streaming is neglected in the analysis of particle acoustophoresis, as the mixed particles were focused on the location away from the channel wall before flowing in the acoustic pressure area. The motion of suspended microparticles in a standing acoustic wave field is primarily determined by the ARF and the Stokes' drag due to the fluid flow [52].

**Fig. 2:**
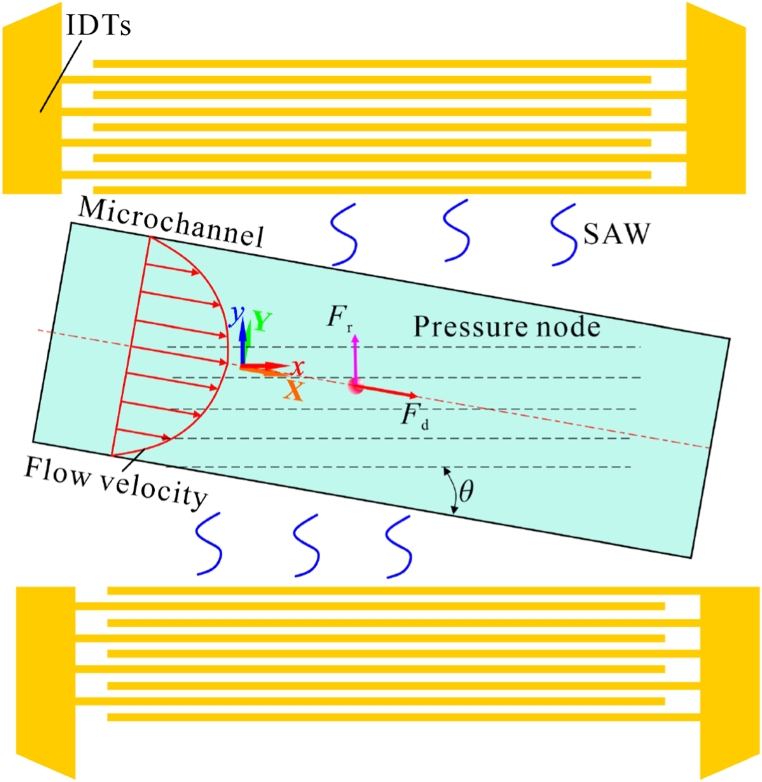
Schematic of the theoretical model for the simulation of particle motion in taSSAW microfluidic device.

As shown in Fig. 2, two coordinate systems were established for the IDTs and the microchannel to facilitate the characterization of the particle motion. The coordinate system of the microchannel is *M*: {*X*, *Y*}, and the coordinate system of IDTs is *N*: {*x*, *y*}. The two coordinate systems can be converted to each other by rotational transformation, and the relationship between them is:

(1) $$\left[\begin{array}{c} x\left(t\right)\\ y\left(t\right) \end{array}\right]=\left[\begin{array}{cc} \cos\;\theta & \sin\;\theta \\ -\sin\;\theta & \cos\;\theta \end{array}\right]\left[\begin{array}{c} X\left(t\right)\\ Y\left(t\right) \end{array}\right]$$

(2) $$\left[\begin{array}{c} X\left(t\right)\\ Y\left(t\right) \end{array}\right]=\left[\begin{array}{cc} \cos\left(\theta \right) & -\sin\left(\theta \right)\\ \sin\left(\theta \right) & \cos\left(\theta \right) \end{array}\right]\left[\begin{array}{c} x\left(t\right)\\ y\left(t\right) \end{array}\right]$$

in the microchannel, particles with a radius *a* suspended in the microchannel with an SSAW field will migrate to the pressure node or the pressure anti-node due to the received ARF. The expression of ARF acting on the particles can be defined as below [53,54]：

(3a) $$F_{\text{rad}}=-\frac{\pi {p_{0}}^{2}V_{p}\beta_{f}}{2\lambda }\varphi \left(\beta ,\rho \right)\sin\left(2ky\right)$$

(3b) $$\varphi \left(\beta ,\rho \right)=\frac{5\rho_{p}-2\rho_{0}}{2\rho_{p}+\rho_{0}}-\frac{\beta_{p}}{\beta_{0}}$$

where *ρ*_p_, *β*_p_, *V*_p_ denotes the density, compressibility, and volume of the particle, respectively; *ρ*_0_ and *β*_0_ denote the density and compressibility of the fluid; *λ* and *k* denote the wavelength and the wavenumber of acoustic wave, respectively; *y* denotes the distance away from pressure node.

Another governing force on the particle is the celebrated Stokes's drag force:

(4) $$F_{d}=-6\pi \mu {av}_{rel}$$

where *μ* is the dynamic viscosity of the fluid and *v*_rel_ denotes the relative particle velocity.

As the operating Reynolds number is relatively low (less than 1), these particles' dynamics in the solvent are overdamped, and the inertial effects can be neglected. In the microchannel coordinate system, the motion of particles can be described by the balance between the ARF and Stoke's drag force along the *X* and *Y* directions [26].:

(5) $$\begin{array}{l} F_{\text{rad}}\;\sin\left(\theta \right)+6\pi \mu a\left(\frac{dX}{dt}-u_{f}\left(Y\right)\right)=0\\ F_{\text{rad}}\;\cos\left(\theta \right)+6\pi \mu a\frac{dY}{dt}=0 \end{array}$$

where *u*_f_ represents the flow velocity where the particle locates.

The expression of Poiseuille flow velocity *u*_f_(*Y*, *Z*) in a rectangular microchannel can be defined by Ref. [55]：

(6) $$u_{f}\left(Y,Z\right)=\frac{4h^{2}\Delta p}{\pi^{3}\mu L}\sum_{n,odd}^{\infty }\frac{1}{n^{3}}\left[1-\frac{\cosh\left(n\pi \frac{Y}{h}\right)}{\cosh\left(n\pi \frac{w}{2h}\right)}\right]\sin\left(n\pi \frac{Z}{h}\right)$$

where *w* and *h* are the width and height of the microchannel, and *L* is the channel length. The SSAW contains standing components along the width direction, resulting in horizontal pressure and velocity profile, and travelling waves moving from the bottom towards the top wall along the height direction, leading to upward-propagating acoustic pressure and velocity [56,57]. The upward travelling SSAW may provide the particles with upward ARF and streaming along the height direction to influence their motion. In this study, we are more concerned with the lateral deflection of particles. Therefore, the influence of upward-propagating wave on particles were neglected. Even though the flow field varies along the height direction, this study uses *Z* = *h*/2 to calculate the flow distribution under different input flow rates for simplification.

Integrating the flow velocity over the microchannel cross-section, the flow rate *Q* can be obtained:

(7) $$Q=2\int_{0}^{w/2}dY\int_{0}^{h}dZu_{f}\left(Y,Z\right)=\frac{h^{4}\Delta p}{12\mu L\text{AR}}\left[1-\sum_{n,odd}^{\infty }\frac{192AR}{{\left(n\pi \right)}^{5}}\tanh\left(\frac{n\pi }{2\text{AR}}\right)\right]$$

where *AR* = *h*/*w* is the aspect ratio of the microchannel *and* Δp is the pressure difference. The total input flow rate *Q* is the sum of the sample flow and the sheath flow.

Combining equations. (1)- (7), supplemented with appropriate boundary conditions, the particle trajectory in taSSAW microfluidic channel can be obtained by solving differential equation (5). We used MATLAB (R2021a, MathWorks) to solve the trajectory of submicron particles. The detailed parameters used in the simulation can be seen in the Supplemental Table S1.

### 2.3 Experimental setup

#### 2.3.1 Design and fabrication of taSSAW microfluidic chip

This study fabricated the microfluidic chip using standard photolithography and mold replica techniques. The microfluidic channel design with a length of 10 mm, width, and height of 800 and 60 μm was fabricated by A 10:1 (weight ratio) Polydimethylsiloxane (PDMS). The IDTs with unidirectional propagation properties were fabricated by lithography and lift-off on a 128° *Y*-cut *X*-propagation lithium niobate substrate. The fabrication process has been previously described [58], and the process can be found in Supplemental Fig. S1. The detailed geometry of the chip design can be found in Fig. S2. The chip and IDTs were bonded after an 80-s O_2_ plasma treatment. The fabricated microchannel and taSSAW chip can be seen in Fig. S3.

The microfluidic chip was mounted on an inverted fluorescence microscope (Nikon Ti2-u, Japan) to observe the state of the particles, and a high-speed CMOS camera (AcutEye 4.0, Rocketech, China) was used to record images during the separation process. A signal generator (AFG31102, Tektronix, USA) was used to provide sinusoidal electrical signals to the transducer to generate SAW. An amplifier (ZHL-5W-1+, Mini Circuits, USA) was used to amplify the signal, and the signal was monitored by an oscilloscope. The sample flow and the sheath flow were injected into the microchannel at a specific flow rate by using the syringe pump (Harvard Pump 11 Elite, USA). The experimental platform detail can be seen in Fig. S4. ImageJ software (NIH, USA) was used for analyzing the experimental images. The network analyzer was used to measure the scattering parameters at the frequency range near the resonance frequency to determine the chip's insertion loss and resonance frequency. To avoid bubble adhering in the microchannel, a syringe pump injected ethanol with low surface energy into the fluidic channel and infiltrated for 5–10 min before the experiment. The flow channel was then rinsed with PBS for 3 min. For biological particle separation, the cleaned microfluidic channel was moistened with 1 % bovine serum protein solution for 5 min. To avoid chip damage and disturbance of flows caused by high temperatures, a real-time semiconductor refrigeration device with TEC-12730 was integrated into the experiment to ensure rapid heat dissipation and refrigeration during the separation process so that the temperature inside the substrate and the microfluidic channel is kept within a constant range.

#### 2.3.2 Sample preparation and exosome characterization

Polystyrene (PS) particles with different diameters (0.1, 0.5 μm) were used for the experiment. The density of polystyrene particles used in the experiment is 1050 kg/m^3^, and the size uniformity difference is less than 5 %. 0.5 wt% Tween 20 was added to the sample solution to prevent particle adhesion and particle interaction. The prepared sample was mixed with a shaker and loaded into a syringe. For exosome separation, supernatant of A549 lung cancer cells and plasma were used. The supernatant of the cultured cells was centrifuged and then filtered to remove particles larger than 0.8 μm. The plasma samples were centrifuged at 10000 g for 30 min to remove cells, cell fragments, and large vesicles and then diluted with PBS buffer. Transmission electron microscopy (TEM) and nanoparticle tracking analysis (NTA) were applied to characterize the morphology and concentration of the exosomes, respectively, and the detail can be found in our previous work [59].

## 3 Results and discussion

This study mainly considers taSSAW microfluidic separation based on size differences. The trajectory of PS particles with a diameter of 0.1, 0.15, 0.2, 0.3, 0.5, and 0.7 μm was simulated and analyzed. The particles were released from *Y* = −50 μm. The overlap width of the IDTs is 6 mm.

### 3.1 Validation of the theoretical model

In order to verify the results obtained from the analytical calculations, it is necessary to compare the predictions with the experimental data. The precision of the model has been investigated by comparing model results with the available experimental data in published work [26,47].

**Fig. 3:**
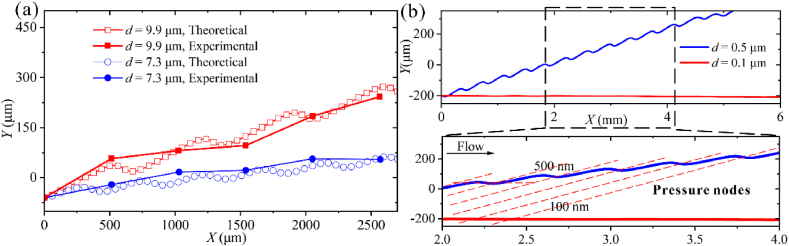
Model validation results. (a) Theoretical and experimental results were measured from published papers for 9.9 and 7.3 μm particle trajectories. (b) The simulated results of 0.5 and 0.1 μm particle trajectories.

The model predictions are compared with experimental data in Fig. 3. For 9.9 μm and 7.3 μm particles [26], the acoustic pressure magnitude is 0.28 MPa. The calculated results almost match the experiments (Fig. 3a). For 500 and 100 nm particles [47], the acoustic pressure magnitude is 5.5 MPa. The simulated trajectories agree with the existing data (Fig. 3b). Considering the uncertain variables of the taSSAW microfluidic separation process in the experiment, the agreement between simulation and experimental results is considered acceptable. We then used the analytical model to study the deflection of submicron-scale particles in taSSAW microfluidic chips.

### 3.2 Influence of acoustic pressure on particle deflection

**Fig. 4:**
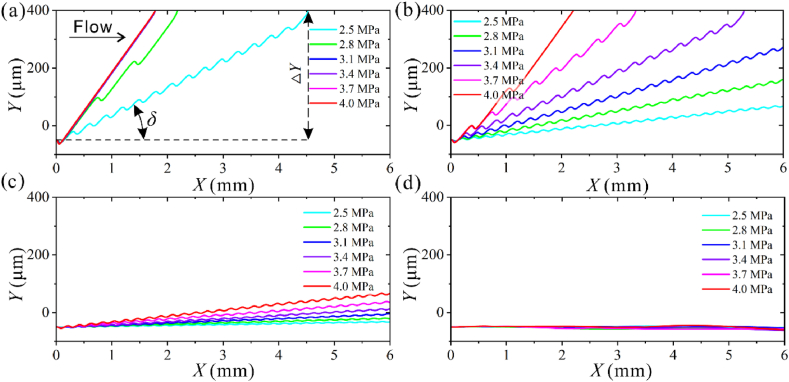
Simulated trajectories of four submicron particles under different acoustic pressure magnitudes with *Q* = 4 μL/min，*f* = 39.97 MHz，*θ* = 15°. (a) *d* = 0.7 μm，(b) *d* = 0.5 μm. (c) *d* = 0.3 μm. (d) *d* = 0.1 μm.

We first calculated the trajectories of four submicron particles under different acoustic pressures with the analytical model. PS particles have a positive acoustic contrast factor and will move toward the wave node. As shown in Fig. 4a, for 0.7 μm particle, when the input flow velocity and other conditions are fixed, the trajectory shifts toward the inlet direction as the acoustic pressure increases, and the angle *δ* between the trajectory and the flow direction keeps increasing. The 0.7 μm particles can be deflected to the side wall of the channel with the acoustic pressure range of 2.5–4 MPa. When *p*_0_ ≥ 3.1 MPa, the particle trajectory coincides with the nodal line in the tilted-angle case, and the maximum value of *δ* equals *θ,* which is a limit for particle deflection.

As shown in Fig. 4b, the 0.5 μm particles are deflected to the nodal line as they enter the acoustic with *p*_0_ = 4 MPa. The particles are deflected to the channel wall in the 3.4–4 MPa acoustic pressure range. When *p*_0_ ≤ 3.5 MPa, the trajectories demonstrate a zigzag shape. For 0.3 μm particles, the maximum lateral deflection distance decreased to 116.7 μm with *p*_0_ = 4 MPa (Fig. 4c). The 0.1 μm particles did not demonstrate apparent deflection in all acoustic pressure ranges (Fig. 4d). For submicron particles, as *p*_0_ increases, *δ* increases, and the effect of ARF is enhanced.

**Fig. 5:**
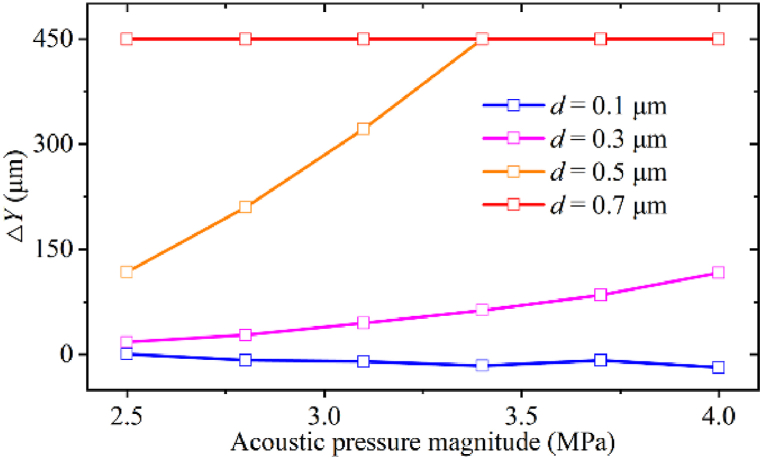
The lateral deflection distance of four particles under different acoustic pressure magnitudes.

The lateral deflection distance Δ*Y* is defined as the distance from its original trajectory (without acoustic field) to the point the particle leaves the acoustic field in the *Y* direction, as shown in Fig. 4a. This parameter Δ*Y* can be used to determine the separation conditions for different mixtures of particles. As shown in Fig. 5, the Δ*Y* of 0.1 μm particles remains almost constant as *p*_0_ increases, and this acoustic pressure range is insufficient to cause the deflection of 0.1 μm particles. Since ARF experienced by particles is proportional to the cube of the particle radius, the ARF dominates over the drag force with increasing *p*_0_. Therefore, the Δ*Y* of 0.3 and 0.5 μm particles increases with *p*_0_. The Δ*Y* of 0.7 μm particles remains constant due to the strong ARF. Depending on the Δ*Y* difference, simultaneous separation of 0.7, 0.5, and 0.3 μm particles can be achieved using 2.5–3.1 MPa acoustic pressure amplitude. To obtain 0.1 μm particles among the four particles, the acoustic pressure should be greater than 3.1 MPa to ensure that all particles larger than 0.1 μm can be deflected sufficiently.

For the taSSAW microfluidic chip, the electrical energy is usually not fully converted to acoustic vibration energy during the excitation of the SAW, resulting in a large amount of heat generation [60]. For submicron-scale particle manipulation, high electrical power is required to ensure that *p*_0_ in the fluid is sufficient to shift the larger particles. The experimentally observed temperature variation at the channel region after the 30s under different input powers is shown in Fig. S5a, where the temperature rises rapidly to 70 °C at a power of 33 dBm. The continuous heat generated by elevated temperatures can heat the fluid, causing bubbles to precipitate, block or disturb the laminar flow. Excessive heat may also have an impact on the activity of the bioparticles. The thermal stress generated by the temperature rise will cause irreversible damage to the lithium niobate substrate [61], such as fractures shown in Fig. S5b. Integrating a real-time cooling device is necessary to avoid the influence of temperature rises on the flow state and chip damage.

### 3.3 Influence of flow rate on particle deflection

**Fig. 6:**
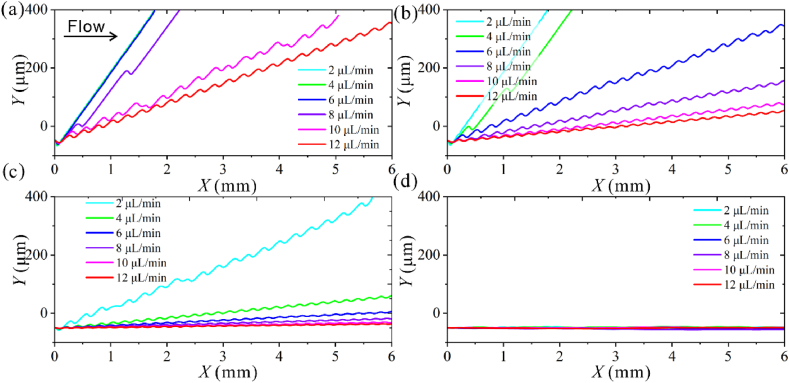
Simulated trajectories of four submicron particles under different acoustic pressure magnitudes with *p*_0_ = 4 MPa, *Q* = 2–12 μL/min, *f*_0_ = 39.97 MHz, and *θ* = 15°. (a) *d* = 0.7 μm. (b) *d* = 0.5 μm. (c) *d* = 0.3 μm. (d) *d* = 0.1 μm.

As shown in Fig. 6a, for 0.7 μm particles, when the acoustic pressure and other conditions are fixed, the particles deflect to the nodal line as they enter the acoustic field with *Q* ≤ 4 μL/min. As the *Q* increases, the trajectory shifts toward the outlet direction. The angle *δ* between the trajectory and the flow direction keeps decreasing because the time particle undergoes in the acoustic field decreases as the *Q* increases. The 0.7 μm particles can be deflected to the channel wall with *Q* ≤ 10 μL/min. As shown in Fig. 6b, the 0.5 μm particles are deflected to the nodal line as they enter the acoustic with *Q* ≤ 2 μL/min. The particles are deflected to the channel wall with *Q* ≤ 4 μL/min. When *Q* ＞ 4 μL/min, the trajectories demonstrate a zigzag shape. The 0.3 μm particles deflect to the channel wall with *Q* = 2 μL/min, and the lateral deflection distance decreased to 112 μm with *Q* = 4 μL/min (Fig. 6c). The 0.1 μm particles did not show obvious deflection in all flow rate ranges (Fig. 6d).

**Fig. 7:**
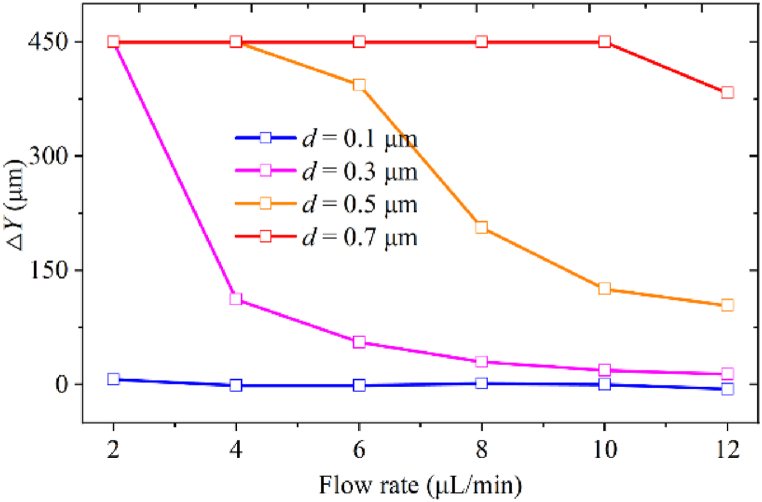
The lateral deflection distance of four particles under different input flow rates.

As shown in Fig. 7, the Δ*Y* of 0.1 μm particles remains constant as *Q* increases, and it is insufficient to cause 0.1 μm particle deflection with *p*_0_ = 4 MPa. A lower flow rate and higher acoustic pressure are required to deflect 0.1 μm particles. The Δ*Y* of 0.3 and 0.5 μm particles decreases nonlinearly with *Q*. The Δ*Y* of 0.7 μm particles remains constant with the flow rate range of *Q* ≤ 10 μL/min due to the strong ARF. Depending on the Δ*Y* difference, simultaneous separation of 0.7, 0.5, and 0.3 μm particles can be achieved with *Q* ≥ 6 μL/min acoustic pressure amplitude. To obtain 0.1 μm particles among the four particles, the input flow rate should be lower than 6 μL/min to ensure that all particles larger than 0.1 μm endure ARF with sufficient time.

Reducing the flow rate is more conducive to deflecting submicron particles. A higher flow rate helps ensure high throughput when meeting the separation efficiency.

### 3.4 Influence of tilted angle on particle deflection

**Fig. 8:**
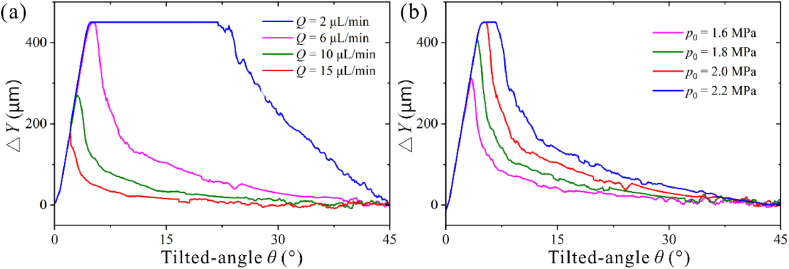
The lateral deflection distance under different tilted angles. (a) 0.7 μm particle at different flow rates with *p*_0_ = 2 MPa. (b) 0.7 μm particle at different acoustic pressure with *Q* = 6 μL/min.

We further studied the lateral deflection distance at different tilted angles *θ*. As shown in Fig. 8a, the △*Y* for 0.7 μm particle increases linearly with *θ* from 0 to a higher value and continuously decreases to 0 with *Q* = 15 μL/min. As flow rates decrease, the peak shifts toward increasing *θ*. The tilted angles corresponding to the maximum deflection distance for the four flow rates are 2.2°, 2.6°, 4.5°, and 4.9–20.5°, respectively. There exists an optimal tilted angle for the 0.7 particle μm to undergo a defined deflection distance, and for example, to achieve △*Y* of 200 μm at *Q* = 6 μL/min, the tilted angle ranges from 2.2 to 7.4°.

As shown in Fig. 8b, as *p*_0_ decreases, the peak shifts toward increasing *θ*. The tilted range for achieving the maximum deflection distance increases with *p*_0_ when particles can deflect to the channel wall. The tilted angles corresponding to the maximum deflection distance for the four flow rates are 3.3°, 4.2°, 4.9–5.3°, and 4.9–6.5°, respectively. The tilted angle *θ* changes the interaction of the ARF and drag force. For high flow rate and low acoustic pressure magnitude, a small tilted angle corresponds to a long ARF acting distance [26,27], thus, the tilted angle to achieve the maximum lateral deflection is reduced. The optimal tilted angle for a single particle can also be obtained by analytical calculation. Similar results for 0.3 μm particles can be seen in Fig. S6.

### 3.5 Influence of SAW frequency on particle deflection

We then investigated the deflections of 0.7 and 0.3 μm particles at different tilted angles using five SAW frequencies of 20, 30,40,50, and 60 MHz. According to the equation *f* = *c*_s_/λ, the resonant frequency can be controlled by adjusting the wavelength of the SAW, and the wavelengths corresponding to the five frequencies are 199.8, 133.2, 99.9, 79.92, 66.6 μm, respectively.

**Fig. 9:**
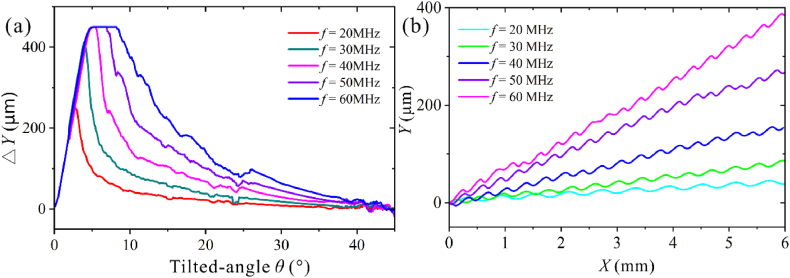
Particle definition under different frequencies. (a) 0.7 μm particle at different flow rates with *p*_0_ = 2 MPa, *Q* = 6 μL/min. (b) The trajectory of 0.7 μm particle at different frequencies with *p*_0_ = 2 MPa, *Q* = 6 μL/min, and *θ* = 10°.

As shown in Fig. 9a, at a specific frequency, the △*Y* for 0.7 μm particles increases, then decreases as the tilted-angle angle increases. As the frequency increases, the tilted angle at which the maximum deflection occurs increases. The tilted angles for the max △*Y* of the five frequencies are 2.7°, 4°, 4.8–5.3°, 4.8–6.7°, and 4.8–8.1°, respectively. The lateral deflection of 0.3 at different frequencies has the same trend. In comparison, 0.3 μm particles require higher acoustic pressure, lower flow velocity, and higher frequency to realize the same deflection as 0.7 μm particles, as shown in Fig. S7.

As shown in Fig. 9b, for 0.7 μm particle, as the frequency increases, the *δ* increases, and its trajectory is shifted toward the inlet. The ARF acting on the particle is proportional to the frequency, so an increase in frequency increases the lateral deflection of the particle. Increasing frequency is more beneficial for generating acoustic energy with higher density [17] and reducing the need for high input power. However, increasing frequency requires lowering the SAW wavelength, which leads to a decrease in the IDT finger's width and increases manufacturing difficulty. When the separation efficiency is met, choosing a minor SAW frequency is more desirable. The increased frequency may result in greater separation. However, the smaller finger width of the IDTs will make it more difficult to manufacture.

### 3.6 Parameter chosen for exosome separation using taSSAW microfluidic chip

We then analyzed the parameters chosen for separate exosome-sized samples from body fluid with particles in the submicron ranges using taSSAW microfluidic chip. The above analysis shows that the tilted angle and SSAW frequency for the design of taSSAW microfluidic chip can be analytically determined, and the flow rate and acoustic pressure in the experiment can regulate the particle deflection. We choose the cell supernatant and human plasma for the sample to obtain exosomes using taSSAW microfluidic chip. The submicron-scale biological particles in human plasma mainly include microvesicles, apoptotic bodies, and exosomes [62]. The diameter range of microvesicles and apoptotic bodies is 150–1000 nm, while exosomes are about 30–150 nm. Analyzing submicron particle deflection is essential to understanding how the acoustic environment affects particle separation and purification.

Since the fluid flow has a Poiseuille distribution, the particle velocity near the wall is close to 0. Therefore, shifting the larger particles to the wall with a high input power is unnecessary. When using the tasSAW microfluidic chip to separate exosomes, it is necessary to ensure that the deflection distance of particles with a size larger than 0.15 μm in the mixed sample is greater than the prefocusing width. Then exosomes with high purity can be obtained by setting appropriate outlets and recycling the treated samples. Based on the analysis mentioned above, we choose the SSAW frequency of 40 MHz, which can provide dense acoustic energy for submicron particle manipulation.

**Fig. 10:**
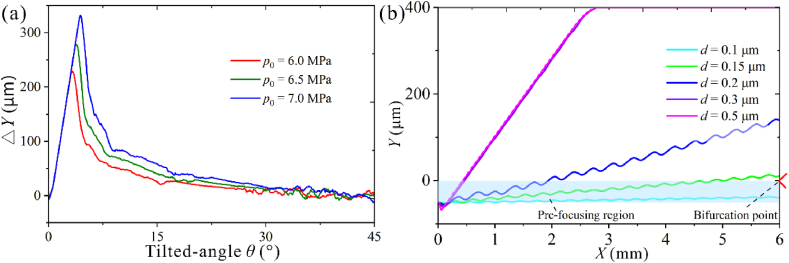
Simulation results for exosome separation. (a) The separation distance of 0.15 μm particles varies with tilted-angle with *Q* = 5 μL/min. (b) The trajectories of submicron mixed particles with *f*_0_ = 40 MHz，*θ* = 10°, and *p*_0_ = 6.5 MPa.

As shown in Fig. 10a, we first calculated the deflection of 0.15 μm particles under different tilted angles. When the prefocus width of the sample is 50 μm, the ideal tilted angles for *p*_0_ = 6.0 MPa, *p*_0_ = 6.5 MPa, and *p*_0_ = 7.0 MPa are 1.1–8.4°, 1.1–13.3° and 1.1–16.3° respectively. We chose *θ* = 10° and calculated the trajectories of 0.1, 0.15, 0.2, 0.3, and 0.5 μm particles with *p*_0_ = 6.5 MPa. As shown in Fig. 10b, the lateral offset distance of 0.15 μm particles is 60.8 μm, larger than the prefocusing width. Except for the 0.1 μm particles, all the submicron particles produced significant lateral deflections. High-purity particles with a diameter smaller than 0.15 μm can be collected below the bifurcation point. Theoretical analysis results show that mixed particle separation efficiency can reach 100 %, similar to the experimental observation [17,63]. We then conducted exosome isolation experiments using taSSAW microfluidic chip based on the analytical analysis on PS particle deflection.

### 3.7 Experimental implementation for PS particles and biological sample manipulation

We fabricated taSSAW microfluidic chips with frequencies of 32.26 MHz and 40 MHz, and both can provide a dense acoustic field to manipulate submicron-scale particles. The S11 scattering parameters of the two chips were analyzed, as shown in Fig. S8, and the operating frequencies were determined from the network analysis.

**Fig. 11:**
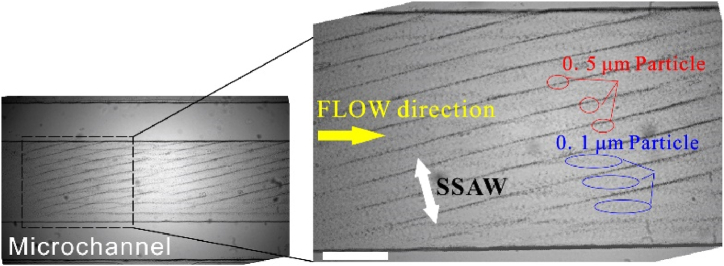
Distribution of 0.5 and 0.1 μm PS particles taSSAW microfluidic channel at an input power of 30 dBm and *f* = 32.26 MHz. The scale bar is 200 μm.

Before the biological sample manipulation, 0.5 μm and 0.1 μm PS particles were injected into the fluidic channel, and the distribution after acoustic wave activation is shown in Fig. 11. Due to the strong ARF, 0.5 μm particles gathered to the nodal lines. In contrast, 0.1 μm particles were still scattered due to the weak ARF. This result demonstrates that a stable SSAW formed in the fluidic channel. The angle between the nodal line and the flow direction is about 15°, which equals the tilted angle of the chip.

**Fig. 12:**
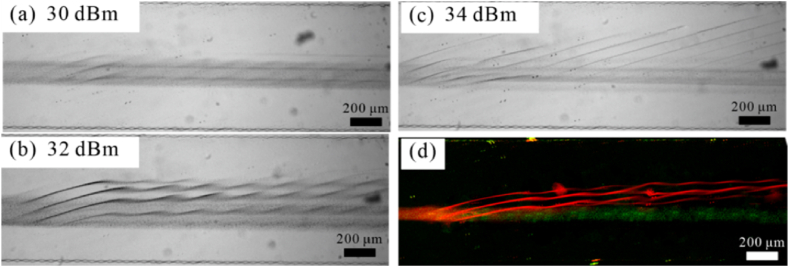
Deflection of 0.5 and 0.1 μm particles under different input powers. (a) 30 dBm; (b) 32 dBm; (c) 34 dBm; (d) 33 dBm. The red color denotes 0.5 μm particle, and the green denotes the 0.1 μm particle. (For interpretation of the references to color in this figure legend, the reader is referred to the Web version of this article.)

The deflection of 0.5 and 0.1 μm particles at different input powers was then investigated with an input flow rate of 5 μL/min, as shown in Fig. 12. When the input power is 30 dBm, a weak deflection of 0.5 μm particles occurs, forming a multi-streamline focusing effect (Fig. 12a). When increasing the power to 32 dBm, the deflection of 0.5 μm particles is more prominent, forming zigzag trajectories (Fig. 12a). At a power of 34 dBm, the 0.5 μm particles shifted toward and moved along the nodal lines. In contrast, the 0.1 μm still moves along the flow direction. At a power of 33 dBm, the trajectories of 0.5 μm particles and 0.1 μm particles were separated in the fluidic channel (Fig. 12d). Submicron particles of high purity were obtained at the exit. By characterizing particle trajectories with fluorescence intensity, the removal efficiency of 0.5 μm particles exceeds 90 %. The experimental results and the analytical calculations agree well.

**Fig. 13:**
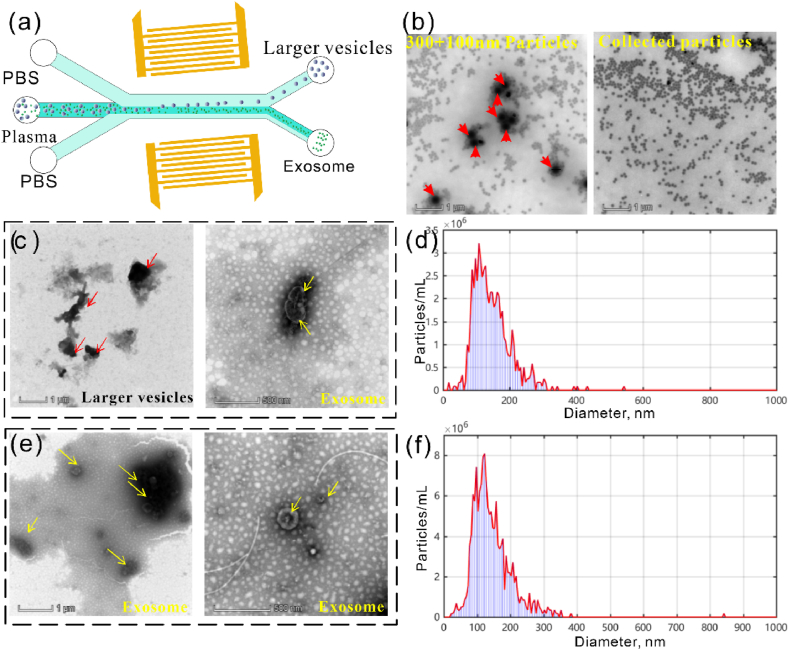
Exosome separation and characterization. (a) Exosome separation chip. (b) TEM results for mixed and collected PS particles. (c) TEM results of larger vesicles and exosomes from the A549 cell supernatant. (d) NTA result of exome separated from the cell supernatant. (e) TEM results of exosome from plasma. (f) NTA result of exome separated from plasma.

We then conducted exosome separation using taSSAW microfluidic chip. The tilted angle was 10° obtained from analytical calculation to ensure that the deflection distance of particles larger than 0.15 μm can exceed the prefocusing length, and the renascent frequency was 40 MHz. The chip structure configuration is shown in Fig. 13a. We first carried out separation experiments of 300 and 100 nm particles, and the flow rates were fixed at 5 μL/min. As shown in Fig. 13b, the mixed particles can be observed before separation, and after manipulation with taSSAW microfluidic chip, purer 100 nm particles can be collected. For the A549 cell supernatant, larger vesicles can be observed with TEM (Fig. 13c), exosomes can be obtained after sorting, the particle size range is comparable to typical exosome sizes 30–150 nm (Fig. 13d). For the plasma sample, after processing with the taSSAW microfluidic chip, exosome samples can be obtained with dimensions comparable to typical exosome sizes (Fig. 13e and f). These results indicate the reliability of the taSSAW microfluidic chip in separating the exosomes.

## 4 Conclusion

In this work, we have systematically investigated the submicron-scale particle acoustophoretic deflection in taSSAW microfluidic chips using an analytical model. The following conclusions may be drawn from the present study:

· The analytical calculations can quickly and accurately solve the trajectories of submicron particles under different acoustic and flow fields.
· From the one-dimensional parameter study, it can be concluded that the submicron particle motion and deflection can be controlled by adjusting the acoustic pressure, fluid flow, SAW frequency, and tilted angle. Increasing the input power or decreasing the input flow helps to achieve greater particle deflection.
· The analytical calculations can help design taSSAW microfluidic chips, such as determining the tilted angle, channel length and resonant frequency.
· Based on the analytical calculation, we carried out exosome sorting from lung cancer cell culture supernatant and plasma samples as an experimental implementation. Larger vesicles at the submicron scale can be effectively removed in taSSAW microfluidic chips to collect exosomes.

In this study, we analyzed particle deflection in taSSAW microfluidic chip based on the size difference. The analytical results in this paper are calculated based on the ideal state. There are still some limitations in this study: it should also be noted that parameters such as compressibility and density also affect particle motion, and isolation of exosome subpopulation subgroups remains challenging [64]. Therefore, in future research, more attention will be paid to the particle behavior with various parameter conditions in taSSAW microfluidic chip to rise to engineer applications. This study may provide a reference for submicron-scale taSSAW microfluidic separation chip design, such as channel parameters (outlet settings) and selection of operational parameters, and may help develop a more efficient, fast, and convenient acoustofluidic separation technology.

## Data availability statement

The data will be available on request.

## CRediT authorship contribution statement

**Tao Peng:** Writing – review & editing, Writing – original draft, Methodology, Investigation, Formal analysis, Data curation, Conceptualization. **Xiaodong Lin:** Methodology, Investigation, Formal analysis. **Luming Li:** Writing – review & editing, Validation. **Lei Huang:** Writing – review & editing, Validation. **Bingyan Jiang:** Project administration, Investigation, Funding acquisition, Formal analysis. **Yanwei Jia:** Writing – original draft, Project administration, Data curation, Conceptualization.

## Declaration of competing interest

The authors declare that they have no known competing financial interests or personal relationships that could have appeared to influence the work reported in this paper.
