# Supplementary material for: Investigation on submicron particle separation and deflection using tilted-angle standing surface acoustic wave microfluidics
Source: Heliyon. 2024 Jan 29;10(3):e25042. doi: 10.1016/j.heliyon.2024.e25042 (PMC10845702; doi:10.1016/j.heliyon.2024.e25042)
Supplement: Multimedia component 1 [file mmc1.zip › Supplemental file.docx]

**Supplemental file**

**Investigation on submicron particle separation and deflection using tilted-angle standing surface acoustic wave microfluidics**

Tao Peng ^1^, Xiaodong Lin ^1^ , Luming Li ^2^, Lei Huang ^2^, Bingyan Jiang ^2*^ , Yanwei Jia ^1,3,4,5*^

^1^ Zhuhai UM Science & Technology Research Institute, Zhuhai, China

^2^ [State Key Laboratory of High-Performance Complex Manufacturing, College of Mechanical and Electrical Engineering, Central South University](https://aip.scitation.org/action/doSearch?field1=Affiliation&text1=State%20Key%20Laboratory%20of%20High-Performance%20Complex%20Manufacturing,%20College%20of%20Mechanical%20and%20Electrical%20Engineering,%20Central%20South%20University&field2=AllField&text2=&Ppub=&Ppub=&AfterYear=&BeforeYear=&access=), Changsha 410083, China

^3^ State Key Laboratory of Analog and Mixed-Signal VLSI, Institute of Microelectronics, University of

Macau, Macau, China

^4^ Faculty of Science and Technology – Electrical and Computer Engineering, University of Macau,

Macau, China

^5^ MoE Frontiers Science Center for Precision Oncology, University of Macau, Macau, China

***Corresponding Author:** jby@csu.edu.cn; [yanweijia@um.edu.mo](mailto:yanweijia@um.edu.mo)

Tab. S1. Material parameters

|  | **Description** | **Symbol** | **Value** |
| --- | --- | --- | --- |
| Water | Density | $\rho_{0}$ | 998 kg/m^3^ |
|  | Speed of sound | $c_{0}$ | 1495 m/s |
|  | Compressibility | $\beta_{0}$ | 4.48×10^-10^ Pa^-1^ |
|  | viscosity | $\mu$ | 0.001 $P\text{a}\cdot\text{s}$ |
| Polystyrene particle | Density | $\rho_{\text{p}}$ | 1050 kg/m^3^ |
|  | Compressibility | $\beta_{\text{p}}$ | 2.49×10^-10^ Pa^-1^ |
| Substrate | Density | $\rho_{\text{s}}$ | 4650 kg/m^3^ |
|  | Speed of sound | $c_{s}$ | 3997 m/s |


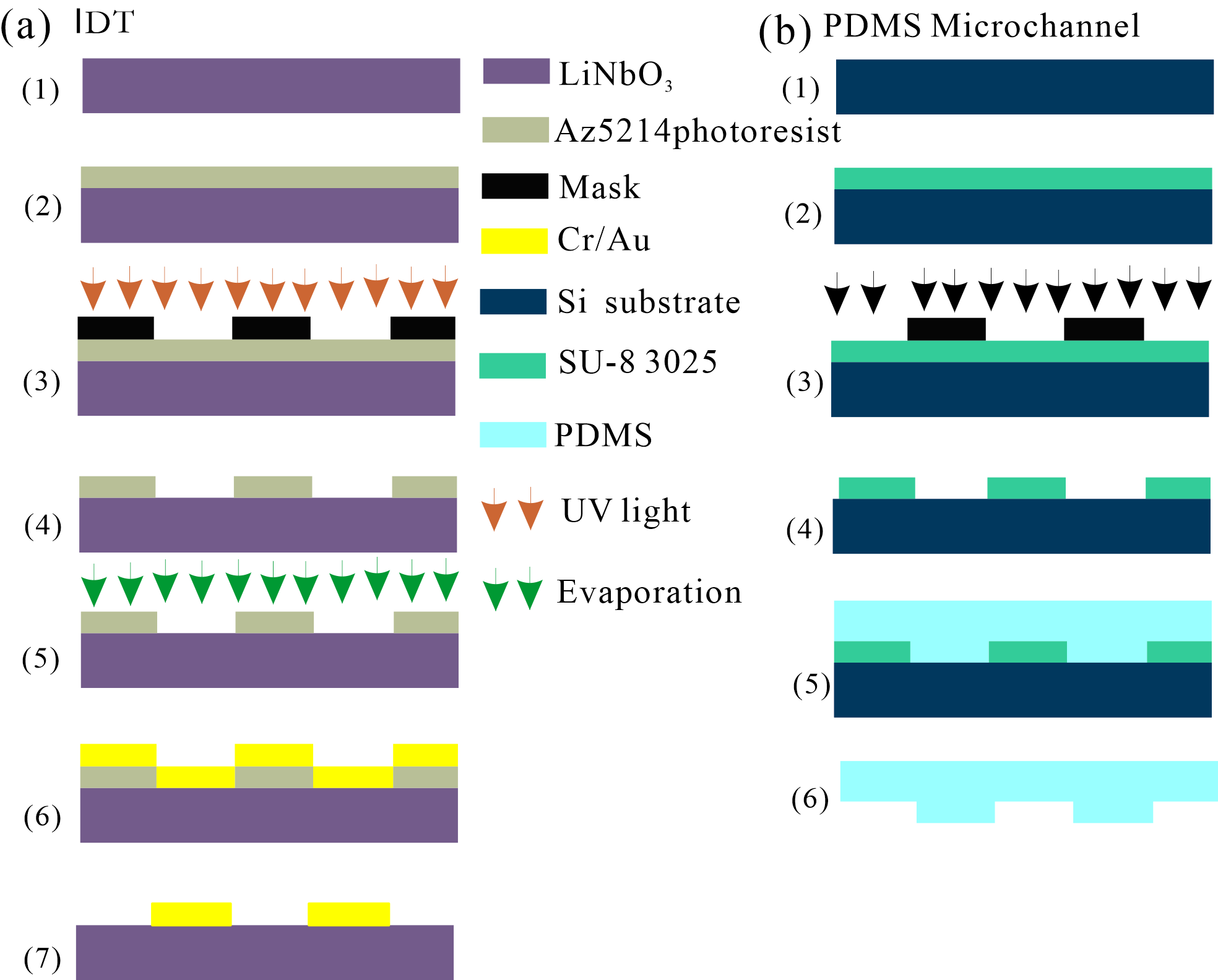


Fig. S1 Fabrication process of taSSAW microfluidic chip. (a) Fabrication process of IDT. (b) The fabrication process of PDMS microfluidic chip.


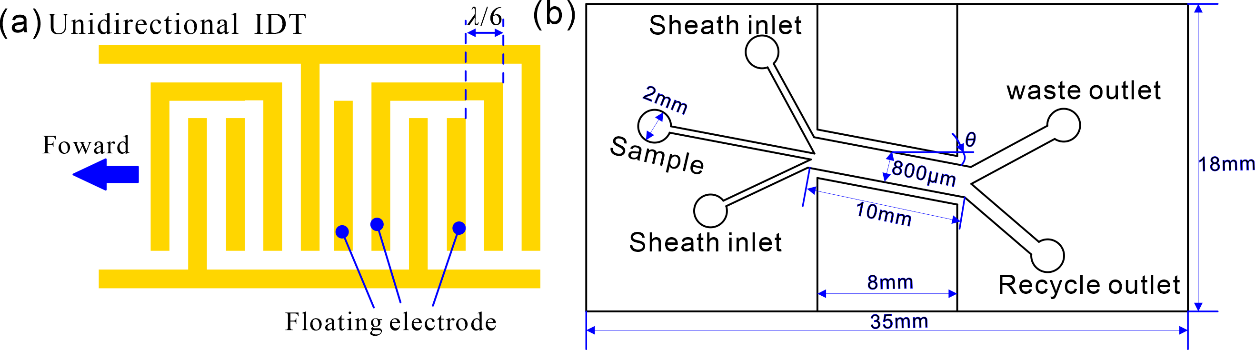


Fig. S2. The schematic design of the microfluidic chip. (a) The unidirectional IDT with floating electrodes. (b) The overall channel design. The flow rate of the upper and lower sheath are 3 and 1 μL/min, the sample flow rate was 1 μL/min.


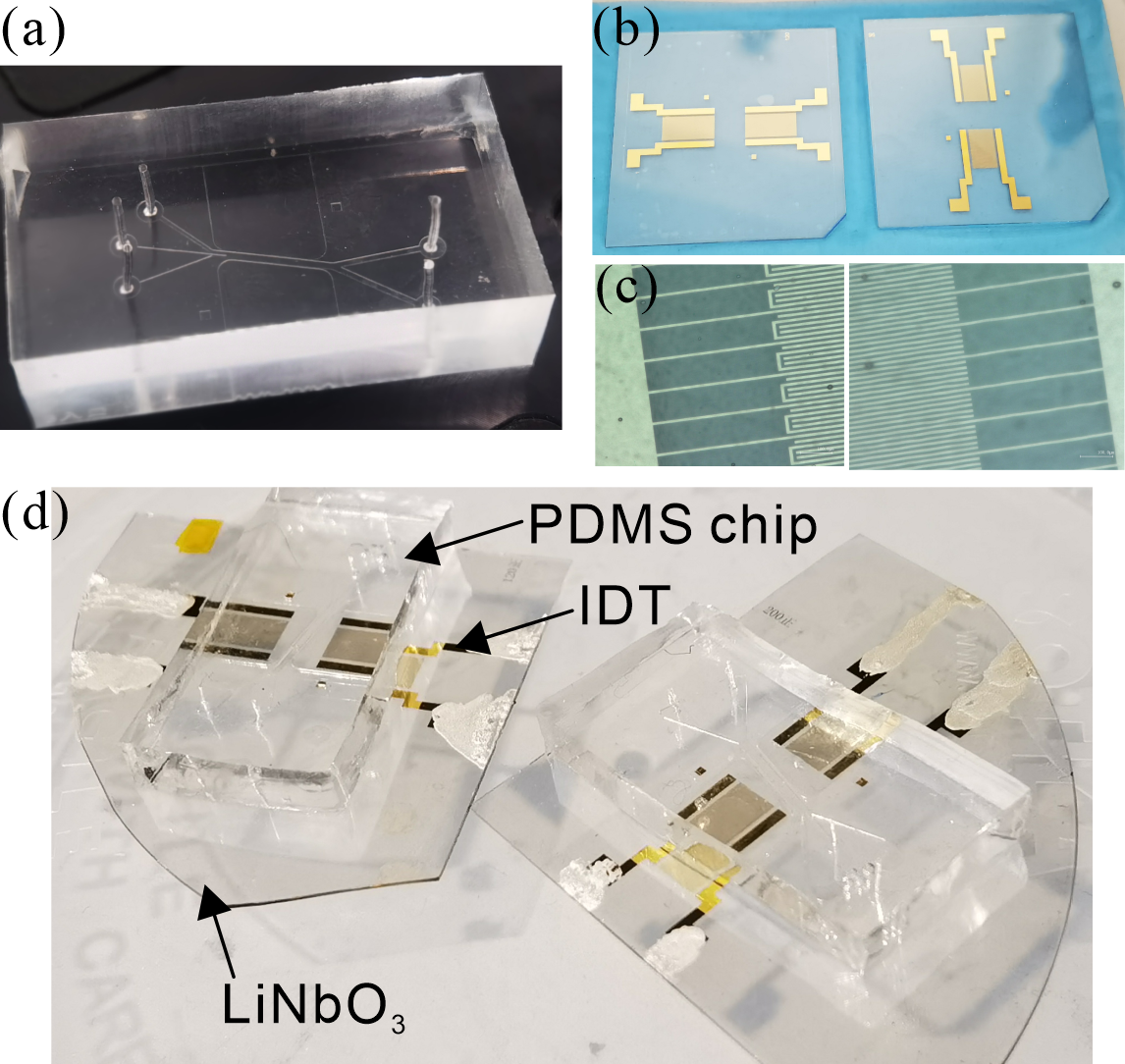


Fig.S3.The fabricated taSSAW microfluidic chip. (a) PDMS chip. (b) IDTs. (c) Detail of the IDT. (d) The assembled taSSAW microfluidic chip.

**
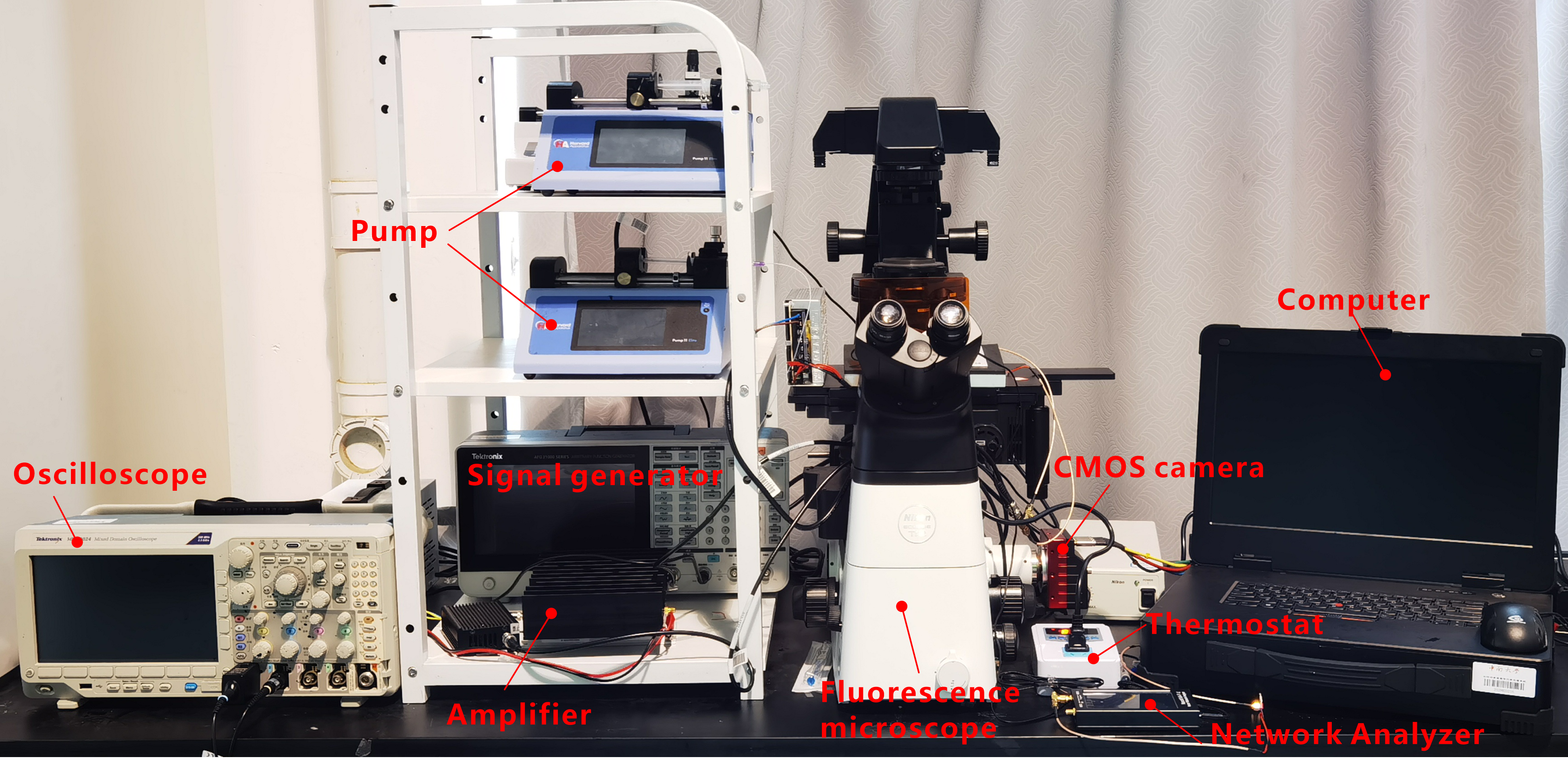
**

Fig. S4. The experimental platform detail.


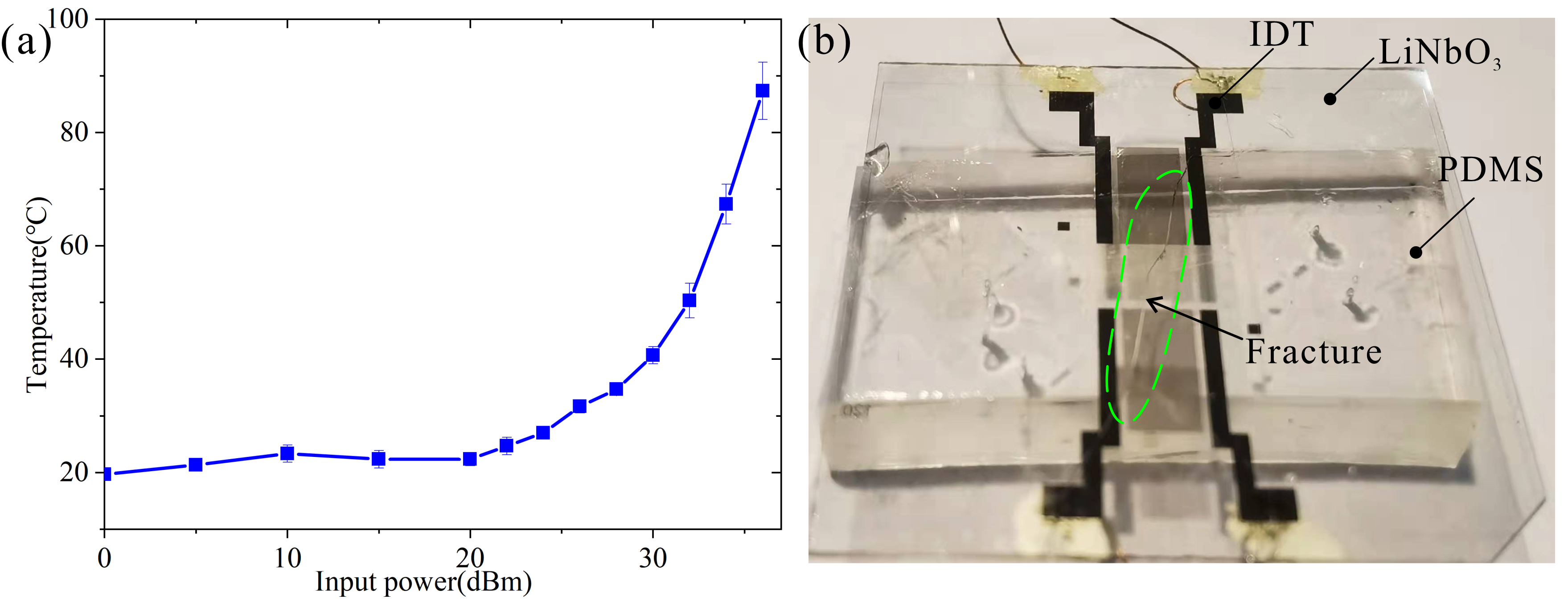


Fig. S5. The temperature rise and fracture of the taSSAW microfluidic chip. (a) Temperature rise under different input powers within 30 s. (b) Chip fracture under an input power of 35 dBm.


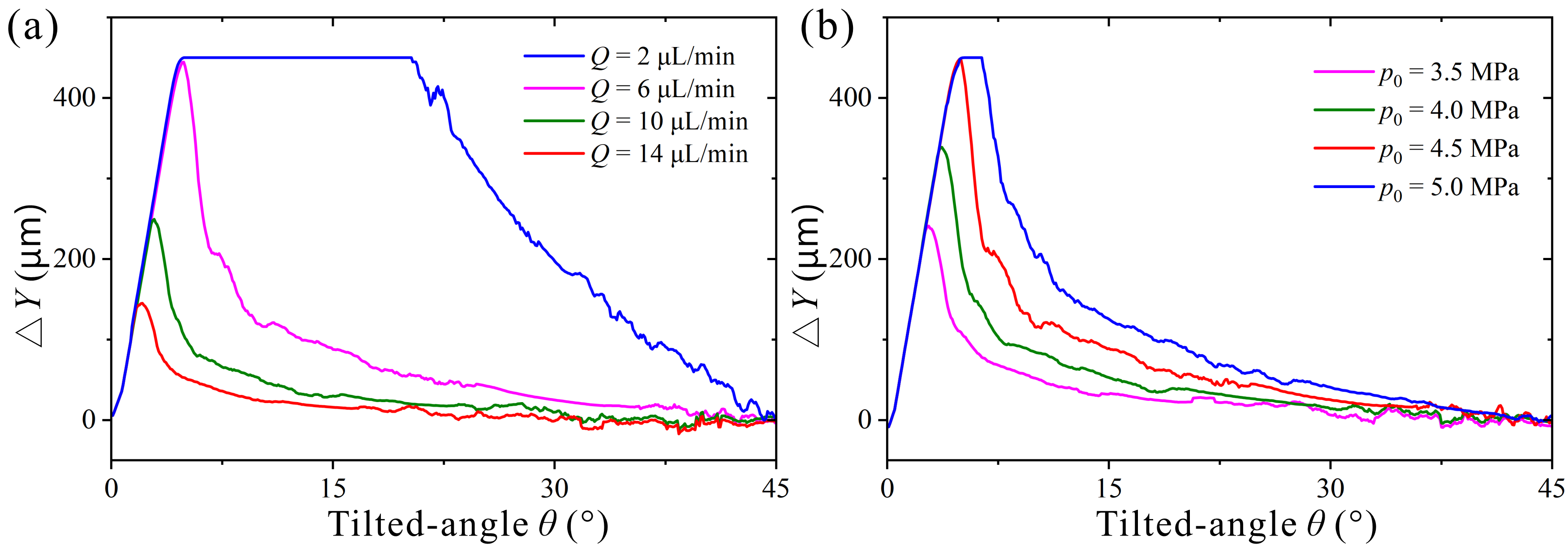


Fig. S6. The lateral deflection distance under different tilted angles. (a) 0.3 μm particle at different flow rates with *p*_0_ = 4.5 MPa. (b) 0.3 μm particle at different acoustic pressure with *Q* = 6 μL/min.


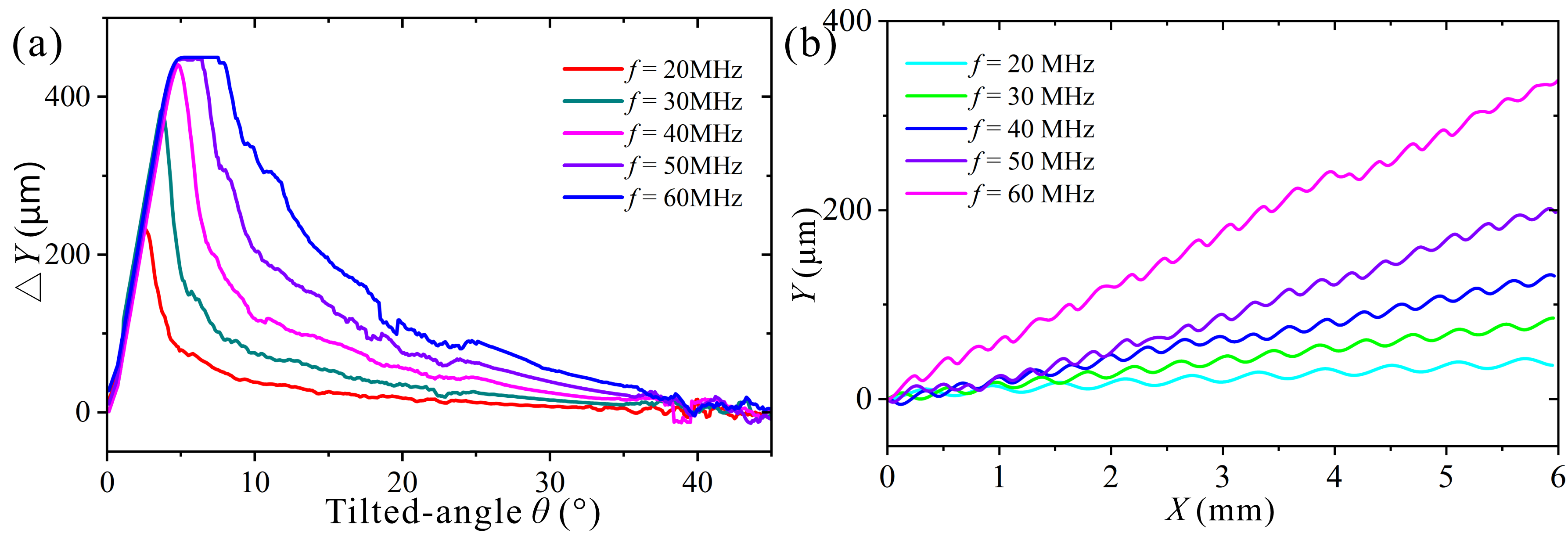


Fig. S7. Particle definition under different frequencies. (a) 0.3 μm particle at different flow rates with *p*_0_ = 4.5 MPa, *Q* = 6 μL/min. (b) The trajectory of 0.7 μm particle at different frequencies with *p*_0_ = 4.5 MPa, *Q* = 6 μL/min and *θ* = 10°.

| 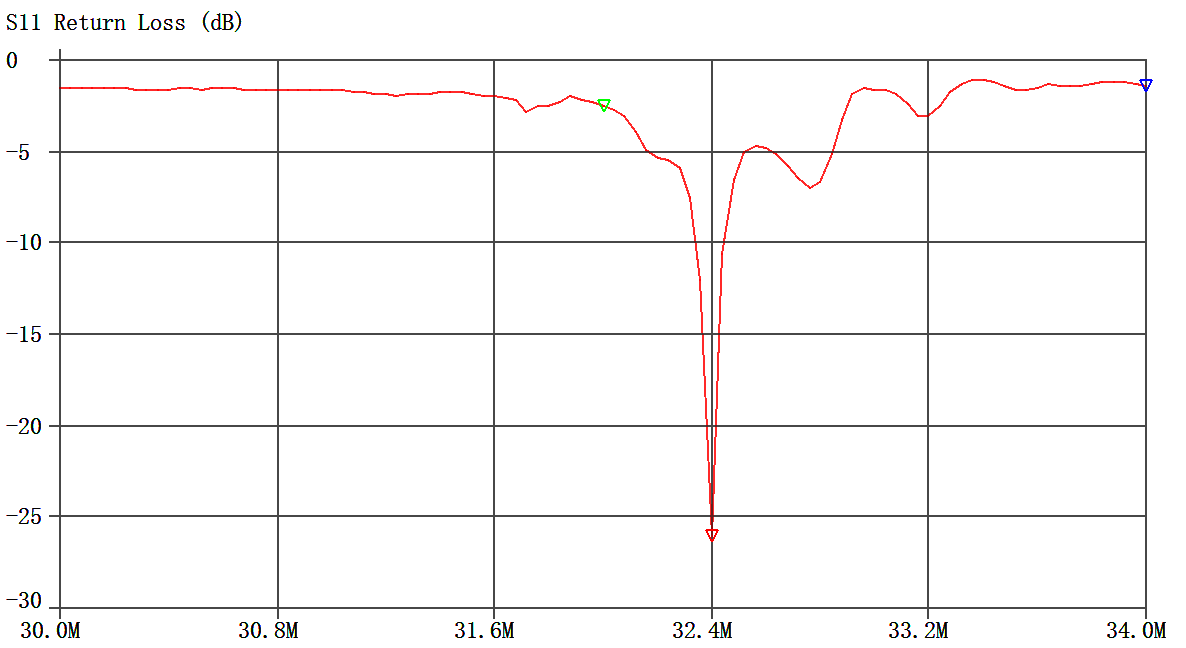 | 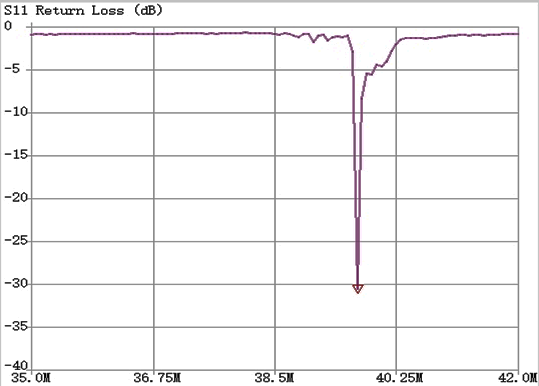 |
| --- | --- |
| (a)*f*_0_ = 32.26 MHz | (b)*f*_0_ = 40 MHz |

Fig. S8. S11 response of IDTs with design frequency of 32.26 MHz and 40 MHz
